# Mucinous Ovarian Cancer in a Young Woman: A Case Report

**DOI:** 10.31729/jnma.9145

**Published:** 2025-07-31

**Authors:** Radhika Kunwar, Beemba Shakya, Karishma Malla Vaidya, Bibhuti Dahal, Shankar Shrivastav

**Affiliations:** 1 Department of Obstetrics and Gynaecology, Paropakar Maternity and Women’s Hospital, Thapathali, Kathmandu, Nepal.; 2 Department of Pathology, Paropakar Maternity and Women’s Hospital, Thapathali, Kathmandu, Nepal

**Keywords:** *completion surgery*, *fertility-sparing surgery*, *mucinous adenocarcinoma of ovary*

## Abstract

Mucinous ovarian cancer is a rare subtype of epithelial ovarian carcinoma, comprising <5% of all cases. Twenty three years old lady with increasing abdominal fullness and discomfort. Contrast enhanced computed tomography revealed a multiloculated ovoid cystic mass (17.5*21.6*9.1)cm in right adnexa. She underwent staging laparotomy with right salphingo-oophorectomy, peritoneal fluid cytology, bilateral pelvic lymph node dissection, infracolic omentectomy and appendectomy. Histopathology confirmed stage IA, grade 1, (well differentiated) mucinous adenocarcinoma of the ovary. After counseling, patient opted for completion surgery and underwent total abdominal hysterectomy with left salphingo-oophorectomy, repeat peritoneal fluid cytology and supracolic omentectomy. Due to malignant cell in the peritoneal fluid, she received adjuvant chemotherapy. She remains disease-free one year postoperatively. Fertility sparing surgery is oncologically permissible for stage IA MOC but contraindicated in 1C3 disease. In Nepal, scaling diagnostic capacity, centralizing services, and integrating culturally sensitive councelling are critical to balance fertility preservation with survival.

## INTRODUCTION

Mucinous ovarian carcinoma (MOC) is a rare variety of epithelial ovarian carcinoma (EOC), with an incidence of <5% among all EOCs.^1^ Most cases are diagnosed at an early stage (stage I) and carry a favorable prognosis. The gold standard operative management involves surgical staging for early disease, while cytoreductive surgery is preferred for advanced disease, however, updated guidelines recommend fertility-sparing surgery (FSS) in selected early stage ovarian cancer cases.^2,3^ Regardless, proper counselling, and shared decision remain key tenets for optimizing outcomes. This report describes a case of MOC managed at Paropakar Maternity and Women’s Hospital.

## CASE

A 23-year-old unmarried nulligravida presented to the outpatient department (OPD) with a 2-month history of progressive abdominal fullness, discomfort, irregular menses, and early satiety. She denied changes in bowel or urinary habits, appetite, weight or night sweats. There was no pertinent medical, surgical or family history of ovarian tumor. Abdominal examination revealed a large, firm, mobile, irregular and nontender mass equivalent to a 34 week gestation, palpable two fingerbreadths below the ribcage.

Ultrasonography (USG) revealed a septated hypoechoic area (15.4 × 8.4 cm) in the right adnexa. Contrast-enhanced computed tomography (CECT) corroborated these finding, demonstrating a multiloculated ovoid cystic lesion (17.5 × 21.6 × 9.1 cm) in the abdominoplevic cavity, originating from right adnexa and extending toward the midline. The lesion exhibited thin and thick septations with an enhancing internal solid component. Mild fluid was noted in the right iliac fossa with preserved fat planes between the bowel, bladder, and rectum, No locoregional lymphadenopathy was observed. The CECT finding were suggestive of mucinous cystadenoma.

Tumor markers were Cancer antigen 125 (CA-125): 306.9 U/mL, Carbohydrate antigen 19-9 (CA 19-9): 6.8 U/mL, lactate dehydrogenase (LDH): 193 U/L, Alpha fetoprotein (AFP) 1.63 ng/mL, Beta-hCG: 0.62 and Carcinoembryonic antigen (CEA): 1.28 ng/mL.

Exploratory laparotomy with intraoperative imprint cytology was planned. Findings included a huge multiloculated right ovarian cyst (24 × 27 × 8 cm) with grossly normal uterus, left ovary, and fallopian tube. Imprint cytology indicated borderline malignancy. Consequently, comprehensive staging laparotomy was performed, comprising right salphingo-oophorectomy, peritoneal fluid cytology, bilateral pelvic lymph node dissection (PLND), infracolic omentectomy and appendectomy.

Final histopathology examination (HPE) reported stage IA, grade 1, well differentiated mucinous adenocarcinoma of the ovary with an unremarkable fallopian tube, appendix, omentum and lymphnode.

**Figure 1:**
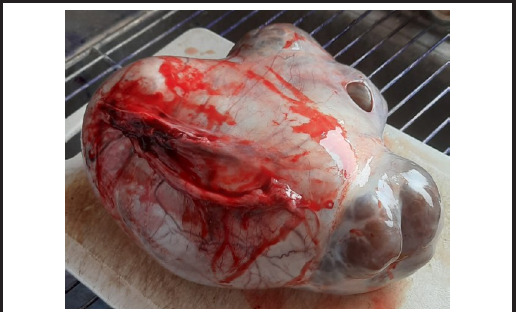
A large right adnexal cyst after being removed from abdomen.

**Figure 2:**
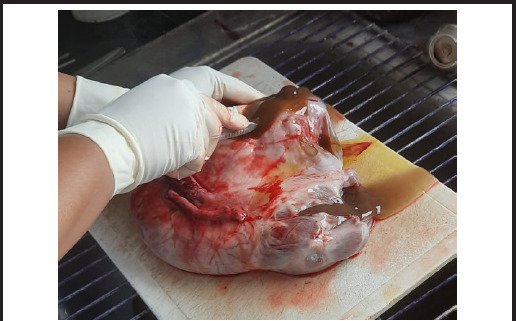
Cross section of the specimen releasing brown viscous fluid.

**Figure 3:**
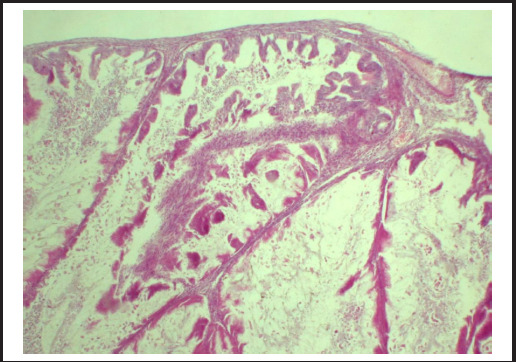
Section from the right ovarian cyst wall reveal tumor cells in back to back as well as fused glands showing expansile growth pattern with minimal intervening stroma.

On Immunohistochemistry, tumor cells showed strong cytoplasmic positivity for CK7 and CK20. Periodic Acid Schiff (PAS) staining was positive in tumor cells. Given a final diagnosis of mucinous adenocarcinoma, the patient and her family were counseled regarding the disease, prognosis and further management. Although fertility sparing surgery had already been performed (appropriate for stage IA grade 1disease) given her unmarried status and desire for future childbearing, the patient and her family opted for completion surgery. Her esophago-gastro-duodenoscopy showed antral gastritis and colonoscopy was normal. Two weeks after the histopathology (HPE) report, completion surgical staging was done, including total abdominal hysterectomy, left salpingo oophorectomy, bilateral pelvic lymph node dissection, supracolic omentectomy and peritoneal fluid cytology. Intraoperative findings included a normal sized uterus adherence of round ligment, and infundibulopelvic ligament to the large bowel and adherence of the left ovary to descending colon. The liver, spleen and undersurface of diaphragm were unremarkable. Peritoneal fluid cytology was positive for malignant cells while histopathology of left ovary, left tube and omentum was unremarkable.

Due to the presence of malignant cells in peritoneal fluid, she was counseled and referred to the clinical oncology department at Bir hospital for chemotherapy. She subsequently received 6 cycles of carboplatin 530mg and paclitaxel 260mg. She remains on regular three-monthly follow-up, with normal tumor markers CA125, CEA, CA19.9 and normal USG abdomen and pelvis reports. She is doing well without evidence of recurrence one year post surgery.

## DISCUSSION

Mucinous ovarian carcinoma is the most common histologic subtype in women under 40 years of age.^4^ Early-stage disease has a favorable prognosis, but advanced stages show poor responsiveness to platinum-based chemotherapy and worse outcomes.^5^

Immunohistochemistry plays a critical role in distinguishing primary MOC from metastases originating in the gastrointestinal tract (e.g., colon, pancreas, and appendix) or tumors of endocervical or endometrial origin. Primary MOC typically exhibits cytoplasmic positivity for CK7+, CK20, while being negative for SABT2, WT1, progesterone, and estrogen receptors.^6^ In this case, strong CK7/CK20 co-expression and periodic acid Schiff (PAS) positivity confirmed primary mucinous adenocarcinoma. Notably, MOC with infiltrative growth patterns carries higher risks of relapse, peritoneal dissemination, lymph nodes involvement, and mortality, whereas the expansible pattern (as observed here) demonstrates minimal stromal invasion and better outcomes.^5^

For early-stage epithelial ovarian cancer (EOC), standard treatment involves hysterectomy with bilateral salpingo-oophorectomy and comprehensive staging. However, unilateral salpingo-oophorectomy with full surgical staging is an option for young women with stage 1A-1C1 disease.^3^ Selection criteria per DiSaia include: stage IA, grade 1 tumors, an encapsulated tumor with no adhesion, no invasion of the capsule, lymphatics, or mesovarium, negative peritoneal washings, and close monitoring.^3^

Recurrence rates after FSS vary by substage, stage 1A, 5-10% recurrence (50% in the contralateral ovary); stage 1C, 1418.6% recurrence (75% extraovarian in 1C3).^7^ Bentivegna et al. reported the long-term outcome of over 500 EOC treated with FSS. The most common subtype was MOC, with 280 patients, with a recurrence rate of 6.8%. The recurrence rate is higher from 9.2% in IC_1_ to 18.6% in IC_3_; furthermore, 75% recurrences were extraovarian after the stage IC_3_ primary tumor. The prognosis of a patient with an extraovarian recurrence is very poor, suggesting that such features could be considered the limits of safety for conservative treatment. It is debatable whether the recurrence was because of preserving the ovary or because of the nature of the disease. The role of FSS should, therefore, be adequately discussed in these patients ^8^

A 2022 meta-analysis (Feng et al.) found FSS safe for stage 1A MOC (5-year survival 94%) but cautioned against its use in 1C3 due to 22% recurrence risk.^9^ ESMO 2023 guidelines stress that FSS requires rigorous patient selection, mandatory completion of surgery after childbearing, and lifelong surveillance ^10^

In Nepal’s resource-constrained setting, FSS faces unique barriers: diagnostic limitations, limited IHC availability impedes accurate MOC diagnosis, risking misclassification of metastatic tumor as primary ovarian cancer. Surgical access: only tertiary centers offer comprehensive staging, rural patient often receive incomplete surgery. Follow-up gaps, poor registry systems and financial barriers reduce adherence to surveillance delaying recurrence detection. Sociocultural factors: early marriage and pronatalist norms heighten fertility-preservation demands, yet inadequate coucelling may prioritize fertility over survival.
